# Transesophageal Echocardiography-Guided WATCHMAN Implantation Without Contrast Use: A Three-Year, Single-Center Experience

**DOI:** 10.7759/cureus.8375

**Published:** 2020-05-31

**Authors:** Muhammad Hamza Saad Shaukat, Hiren Patel, Rizwan Alimohammad, Augustin DeLago

**Affiliations:** 1 Internal Medicine, Albany Medical College, Albany, USA; 2 Cardiology, Albany Medical College, Albany, USA; 3 Cardiology, Capital Cardiology Associates, Albany Medical Center, Albany, USA

**Keywords:** watchman, left atrial appendage occlusion without contrast use, transesophageal echocardiography

## Abstract

Background

It is unclear if the WATCHMAN device (Boston Scientific, St. Paul, Minnesota) can be implanted without contrast to prevent complications in patients with advanced chronic kidney disease (CKD) or contrast allergy.

Objective

The efficiency and safety of WATCHMAN implantation under transesophageal echocardiography (TEE)-guidance and fluoroscopy without contrast use.

Methods

This was a retrospective single-center study at Albany Medical Center between June 2016 and June 2019. Consecutive procedure notes for all WATCHMAN devices implanted between June 2016 and June 2019 were screened to identify patients who did not receive contrast. Patients with incomplete information on the calculation of the 'Congestive heart failure, Hypertension, Age [>75 years], Stroke, Diabetes mellitus, Vascular disease, Age [65 to 74 years], Sex category' (CHA2DS2VASc)/'Hypertension, Abnormal renal/liver function, Stroke, Bleeding history, Labile international normalized ratio, Elderly, Drugs/alcohol' (HAS-BLED) score and reason(s) precluding safe contrast use were excluded. Efficiency was measured as i) accuracy of device size estimation based on TEE-measured left atrial appendage (LAA) dimensions were determined by the need to change the size of the device initially selected, ii) number of implantation attempts, irrespective of change in device size, iii) whether more than one device was used secondary to inaccurate initial size estimation or other procedural complexities, and iv) successful LAA seal on TEE immediately and 45-days post-implantation (peri-device leak of < 5 mm by color Doppler). Procedure-related complications, immediate and delayed (0-45 days), were recorded.

Results

Twelve patients received WATCHMAN without contrast. The mean age was 79.2 years, with male predominance (n=8). The mean CHA2DS2VASc and HAS-BLED scores were 5.50 (+/-1.24) and 4.08 (+/-1.08), respectively. Contrast was avoided because of a history of CKD stage IV (n=5), rapidly progressive CKD stage III (n=1), and contrast allergy (n=6). In 11 out of 12 patients, initial TEE-based device size estimation was accurate with successful implantation at the first attempt. One patient required a change in initial device size and, therefore, required a second attempt for successful implantation. There was no peri-device leak immediately post-implantation in any patient; only one patient had a significant device leak on day 45 TEE requiring continuation of anticoagulation for four months until a successful device seal. There were no immediate or late complications up to 45-days post-implantation.

Conclusion

Our experience shows no significant compromise in the efficiency and safety of the WATCHMAN implantation without contrast in patients with advanced CKD or a contrast allergy.

## Introduction

Since the Prospective Randomized Evaluation of the Watchman LAA Closure Device In Patients With Atrial Fibrillation Versus Long Term Warfarin Therapy (PREVAIL) study demonstrated its noninferiority to warfarin for thromboembolism prevention in non-valvular atrial fibrillation, left atrial appendage (LAA) occlusion is increasingly offered to patients who are unable to tolerate long-term anticoagulation [1]. Intraprocedural real-time transesophageal echocardiographic (TEE) and fluoroscopic imaging are used to visualize anatomic structures, catheters/guidewires, and LAA occlusion devices [2]. Fluoroscopic guidance, in addition to radiation exposure, involves the use of nephrotoxic contrast. Over one-third of patients with an implanted LAA occlusion device have chronic kidney disease [3]. We report our experience performing LAA occlusion with the WATCHMAN (Boston Scientific, St. Paul, Minnesota) device, guided by TEE and fluoroscopy without contrast administration.

## Materials and methods

Consecutive procedure notes for all WATCHMAN devices implanted between June 2016 and June 2019 were screened to identify patients who did not receive contrast. The electronic records of these patients were reviewed for demographic information; comorbid conditions; baseline renal and liver function; 'Congestive heart failure, Hypertension, Age [>75 years], Stroke, Diabetes mellitus, Vascular disease, Age [65 to 74 years], Sex category' (CHA2DS2VASc) and 'Hypertension, Abnormal renal/liver function, Stroke, Bleeding history, Labile international normalized ratio, Elderly, Drugs/alcohol' (HAS-BLED) scores; reason(s) precluding safe use of contrast; and additional risk factors for bleeding or thrombosis not captured by the CHA2DS2VASC/HAS-BLED scores. Patients lacking complete information to calculate the CHA2DS2VASc/HAS-BLED score were excluded.

The following endpoints were assessed:

a. Efficiency i) accuracy of device size estimation based on TEE-measured LAA dimensions was determined by the need to change the size of the device initially selected ii) number of implantation attempts, irrespective of change in device size iii) whether more than one device was used secondary to inaccurate initial size estimation or other procedural complexities iv) successful LAA seal on TEE immediately and 45-days post-implantation (peri-device leak of < 5 mm by color Doppler)

b. Safety-procedure-related complications, immediate and delayed (0-45 days)

Microsoft Excel (Microsoft Corporation, Redmond, Washington) was used for statistical analysis. Continuous variables are expressed as mean with standard deviation (mean +/- SD); categorical variables as percentages.

## Results

**Figure 1:**
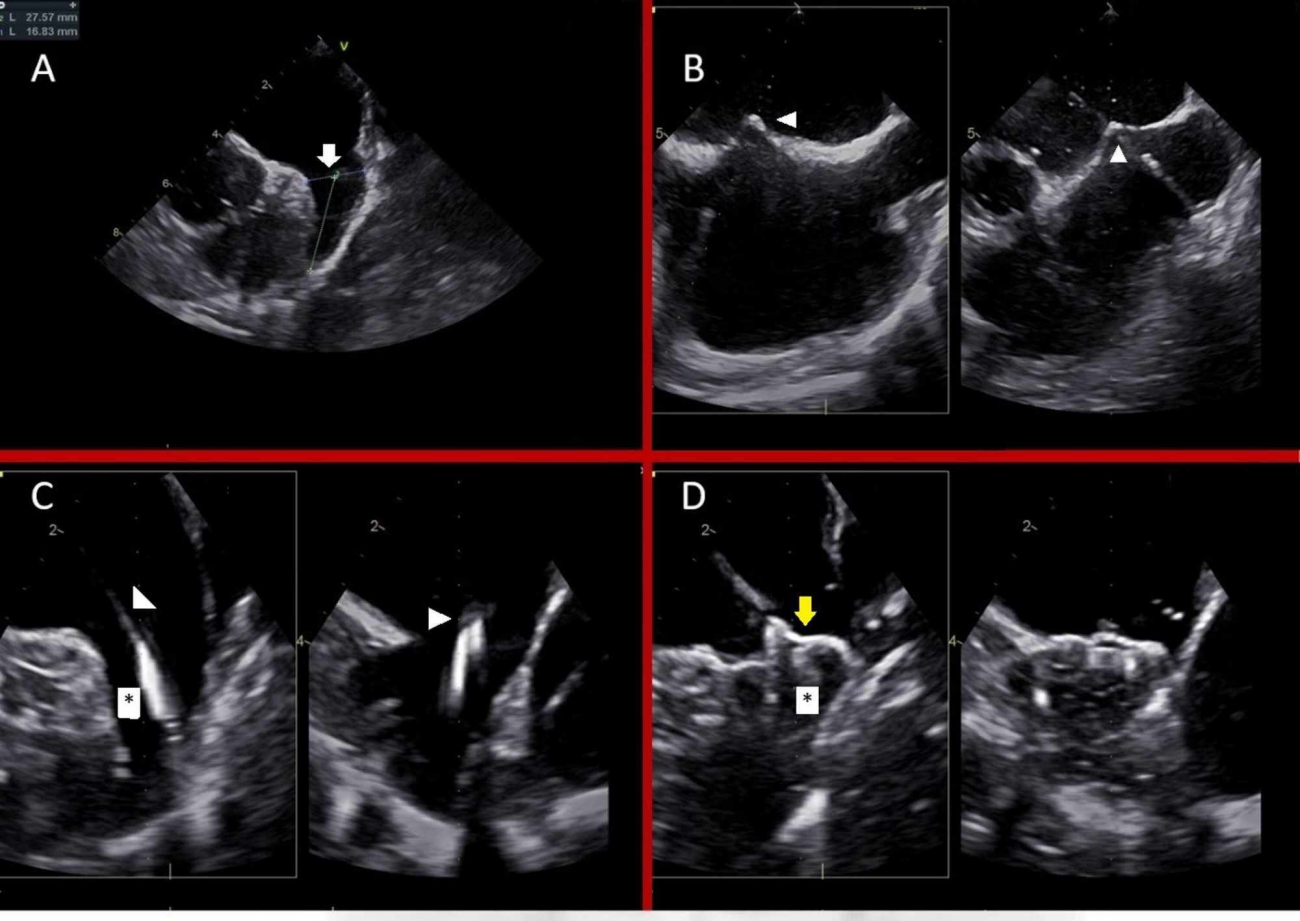
TEE-guided WATCHMAN implantation Panel A: LAA measurement on appendage view (arrow). Panel B: Bicaval view showing tenting of the interatrial septum (arrowheads). Panel C: Appendage view: Delivery catheter (arrowheads) in LAA (asterisk). Panel D: WATCHMAN (yellow arrow) implanted in LAA (asterisk). TEE: Transesophageal echocardiography; LAA: Left atrial appendage WATCHMAN: Boston Scientific, St. Paul, Minnesota

The WATCHMAN device was implanted in 13 patients under TEE and fluoroscopic guidance only without any contrast use (Figures 1A-1D).

One patient was excluded because of incomplete information to calculate the CHA2DS2VASC/HAS-BLED scores. The final study cohort consisted of 12 patients with a mean age of 79.2 years and male predominance (Table 1).

**Table 1:** Baseline characteristics a Myocardial infarction, peripheral vascular disease, aortic plaque; b Total bilirubin > twice upper-limit of normal or liver function tests >3 times upper limit of normal; c Serum Cr >2.2 mg/dl, renal transplant or end-stage renal disease on hemodialysis TIA: transient ischemic attack; eGFR: estimated glomerular filtration rate; CHA2DS2VASc: Congestive heart failure, Hypertension, Age [>75 years], Stroke, Diabetes mellitus, Vascular disease, Age [65 to 74 years], Sex category; HAS-BLED: Hypertension, Abnormal renal/liver function, Stroke, Bleeding history, Labile international normalized ratio, Elderly, Drugs/alcohol

Baseline characteristics	n=12 (%)
Mean age (years +/- SD)	79.2 +/- 5.44
Female	4 (33.3)
Congestive Heart Failure	7 (58.3)
Hypertension	11 (91.7)
Diabetes Mellitus	5 (41.7)
Stroke/TIA/Venous Thromboembolism	7 (58.3)
Vascular Disease ^a^	5 (41.7)
Abnormal Liver Function ^b^	0 (0)
Abnormal Renal Function ^c^	3 (75.0)
Mean eGFR [ (ml/min/1.73 m^2^) +/- SD]	46 +/- 17.4
Mean CHA2DS2VASc score +/- SD	5.50 +/- 1.24
Mean HAS-BLED score +/- SD	4.08 +/- 1.08

The mean CHA2DS2VASc and HAS-BLED scores were 5.50 (+/-1.24) and 4.08 (+/-1.08), respectively. Reasons for LAA occlusion with WATCHMAN included non-variceal gastrointestinal bleeding requiring blood transfusions (n=5), recurrent gastroesophageal variceal bleeding (n=2), and worsening hematuria in the setting of urothelial cancer (n=1). Four patients received WATCHMAN to prevent bleeding associated with high fall risk: Elderly on triple anti-thrombotic therapy (n=2), uncontrolled seizures (n=1), and advanced Parkinson’s disease (n=1).

Contrast use was avoided because of chronic kidney disease stage IV (n=5), rapidly progressive chronic kidney disease stage III (n=1), and contrast allergy (n=6).

Mean LAA depth and ostial diameter were 27.9 +/- 5.70 mm and 20.5 +/- 5.03 mm, respectively. THE mean WATCHMAN device size was 28.0 +/- 4.11 mm. In 11 out of 12 patients, initial TEE-based device size estimation was accurate with successful implantation on the first attempt (Table 2).

**Table 2:** Procedural efficiency ^a ^<5 mm leak on Doppler ultrasound WATCHMAN: Boston Scientific, St. Paul, Minnesota

Procedural Efficiency	n=12 (%)
Accurate initial WATCHMAN size estimation	11 (91.7)
WATCHMAN implantation successful on initial attempt	11 (91.7)
Single device used	11 (91.7)
Successful device seal immediately post-implantation ^a^	12 (100)
Successful device seal 45 days post-implantation ^a^	11 (91.7)

One patient required a change in initial device size and, therefore, required a second attempt for successful implantation. There was no peri-device leak immediately post-implantation in any patient; only one patient had significant device leak on day 45 TEE, requiring continuation of anticoagulation for four months until successful device seal on repeat TEE. There were no immediate or late complications up to 45 days post-implantation (Table 3).

**Table 3:** Procedural complications ^a ^Severe bleeding: hemodynamic instability, end-organ dysfunction, or requiring transfusion WATCHMAN: Boston Scientific, St. Paul, Minnesota

Procedural Complications	n=12 (%)
Cardiac tamponade	0 (0)
Pericardial effusion/pericarditis	0 (0)
Device embolization/stroke/death	0 (0)
Severe bleeding on post-WATCHMAN anticoagulation ^a^	0 (0)
Non-severe bleeding on post- Watchman anticoagulation	1 (8.33)

## Discussion

In 11 out of 12 patients, TEE without contrast fluoroscopy accurately measured ostial diameters and the depth of the LAA. This translated into appropriate WATCHMAN size estimation and successful implantation on the initial attempt. Only one patient required a change of device due to incorrect initial WATCHMAN size estimation (correctly re-measured with echocardiography). There were no periprocedural complications up to 45 days post WATCHMAN.

There are limited data on LAA occlusion guided solely by TEE. A single-center prospective study with 14 patients demonstrated successful and safe implantation of LAmbre (LAA occlusion device; Lifetech Scientific (Shenzhen) Co. Ltd., Shenzhen, China) with TEE only [4]. There is a single case report of TEE-guided WATCHMAN implantation without contrast in an elderly patient with advanced chronic kidney disease, similar to the patients reported in our study [5]. About 40% of patients with an LAA occlusion device are estimated to have chronic kidney disease [3]. Patients with chronic kidney disease are at increased risk of both bleeding and thromboembolism. Evidence for direct-acting oral anticoagulants (DOACs) in chronic kidney disease is derived, at best, from post-hoc analyses. LAA occlusion has shown comparable efficacy in stroke prevention in patients with or without chronic kidney disease [6-8]. Avoiding contrast in LAA occlusion procedures offers the additional benefit of reducing the risk of contrast-associated acute kidney injury [9]. Notwithstanding its dependence on the acquisition of high-quality 3-D TEE images, our study suggests that contrast during WATCHMAN implantation may be avoided without compromising the efficiency or safety of the procedure.

The limitations of our study are acknowledged. Although it is, to our knowledge, the largest study to-date on WATCHMAN implantation without contrast, the sample size is small and limited to a single center. Due to the retrospective nature of the study, it is unclear how the technique affects radiation exposure and procedure time. A direct comparison of WATCHMAN implantation with and without contrast is needed.

## Conclusions

This is the largest reported, single-center study on successful LAA occlusion with the WATCHMAN device under TEE and fluoroscopy guidance without the use of contrast. Avoiding contrast, in our experience, does not significantly compromise the efficiency and safety of the procedure in patients with advanced CKD or contrast allergy.
